# Essays on Hypochondriacal and Other Nervous Affections

**Published:** 1816-09

**Authors:** 


*Essays on Hypochondriacal and other Nervous Affections.* By John Reid, M.D. Member of the Royal College of Physicians, London; and late Physician to the Finsbury Dispensary. 8vo. pp. 272. London, 1816.

So close is the connexion of the mind and the body, and such is their mutual influence in action or suffering, that in no possible case of human life, can the one be affected without producing some impression on the feelings of the other. Yet this truth, which has never been denied even by the sternest stoic, or the most subtle metaphysician, has attained universal assent, rather as an article of sensation than of science; as being the compulsion of experience, more than the result of enquiry. Some men may call in question the liberty of the human will; and others may weave a fine spun chain of arguments against the existence of matter and the reality of an external world: but after all, whether our thoughts are strung together by necessity, and our movements are impelled by causes over which we have no controul; whether the forms that occasion pleasure or pain are substantial or ideal, still the conclusion is the same, as it relates to the sympathy by which the mental and corporeal faculties mutually operate to the ease or disquiet of the entire system. It is, therefore, mortifying to the pride of man's wisdom, that in an age which, above all others, has pushed with the greatest effect the energy of philosophical investigation into the hidden mysteries of Nature, which has traced the minutest essences through a variety of modifications to their elemental principles, with an assiduity that claims applause, and a success that commands admiration; still, after all, and to abate the swell of vanity arising from this consciousness of intellectual superiority, man is made to confess that he knows more of the world around him, than of that which he carries within his own compact form. They who, more than others, are obliged to study the constitution of the human frame, and to extend their inquiries into a vast variety of scientific objects, that they maybe qualified to render that study complete and beneficial, even the professors of the noblest art that can engage the time and the talents of man, are in like manner compelled to acknowledge their total inefficiency to account for the aberrations that so frequently disturb the machine with which they are most acquainted. This *crux medicorum* is the more distressing, because while it urges application, it confounds the judgement; and at the same time that it calls for the discovery of a remedy, it baffles hope, in being under the necessity of leaving that to chance which can never be determined by any certain principle of operation, or be regulated by any rule of practice. Without presuming to advance that the awful malady of Insanity, in all its shades of gradation, can never become so precisely defined as to admit of medical management with a probability of success; we must at least be allowed to say, that hitherto our acquaintance with mental diseases has gone little further than to an observation of general causes and effects, over which professional skill may exert its best efforts in vain. With this impression on our minds, we took up the present volume, in the expectation of seeing much ingenuity wasted in an attempt to remove that opprobrium of medical science, which the subject of insanity has so long proved. We were prepared, indeed, from what we had with pleasure read in the periodical reports of the Author, to meet with some acute and lively remarks on extraordinary cases, as also with sagacious counsel in regard to the means employed for the relief of persons affected by nervous disorders. But we had no thought that in a book bearing an unassuming title, and upon an unpromising topic, would be found so many new lights, in the simple form of hints, on the various excitements to mental disease, and upon the injudicious manner in which they are too commonly treated. That the Author has been prevented from fulfilling his original intention of publishing a systematical treatise on the subject, is rather a matter of congratulation than otherwise; as, by throwing out his observations in the form of Essays, he has rendered his work more likely to become popular and beneficial, by bringing it immediately to the view of those who would be alarmed at the thoughts of perusing any thing like a theoretical or argumentative performance.

The Essays are twenty-seven in number; and although they have not the formality of a connected arrangement, yet, as if the maxim of Horace had been under cont mplation, the reader will find in his progress, that the order could not have been better disposed, even to constitute a train of leading principles.

The first Essay is "On the Influence of the Mind on the Body," in which consciousness, as the peculiar faculty of man, is set forth in strong and elegant language. The following remark, at the close, strikes us as equally new and important.

"The class of persons whose lives are devoted to mere manual labor, especially the more indigent part of them, are, to a certain extent, distinguished by the character of their diseases, as well as that of their other evils. They differ from the higher orders, less perhaps in the actual quantity, than in the glaring and obtrusive color of their calamities.

"There is no person, perhaps, who is apt to form so low an estimate of the value of human existence, as a medical man practising amongst the poor, especially amongst the poor of a great city. But it is not impossible that he may exaggerate the excess of their sufferings, by combining, as it is natural for him to do, their external state with those feelings which he has acquired from very different circumstances and education. As the horrors of the grave affect only the living, so the miseries of poverty exist principally, perhaps, in the immagination of the affluent. The labour of the poor man relieves him at least from the burden of fashionable ennui, and the constant pressure of physical inconveniences, from the more elegant, but surely not less intolerable distresses of a refined and romantic sensibility. Even those superior intellectual advantages of education, to which the more opulent are almost exclusively admitted, may, in some cases, open only new avenues to sorrow. The mind in proportion as it is expanded, exposes a larger surface to impression."

Essay II. On "The Power of Volition," exhibits some curious cases, wherein several persons have been known to "possess power not only over the feelings and faculties of the mind, but likewise over what are called the involuntary muscles, and even the blood vessels of the body. But, amusing as this part of the Essay is, what the Author has observed on the inhumanity of treating hypochondriacs with ridicule, is better adapted to improve the feelings and to regulate the practice of men.

"No one was ever laughed or scolded out of hypochondriasis. It is scarcely likely that we should elevate a person's spirits by insulting his understanding. The malady of the nerves is in general of too obstinate a nature to yield to a sarcasm or a sneer. It would scarcely be more preposterous to think of dissipating a dropsy of the chest, than a distemper of the mind by the force of ridicule or rebuke. The hypochondriac may feel indeed the edge of satire as keenly as he would that of a sword; but although its point should penetrate his bosom, it would not be likely to let out from it, any portion of that noxious matter by which it is so painfully oppressed. The external expression of his disorder may be checked by the coercive influence of shame or fear; but in doing this, a similar kind of risque is incurred as arises from the repelling of a cutaneous eruption, which, although it conceal the outward appearance, seldom fails still more firmly to establish the internal strength, to increase the danger, and to protract the continuance of the disease. By indirect and imperceptible means the attention may, in many instances, be gently and insensibly enticed, but seldom can we with safety attempt to *force* it from any habitual topic of painful contemplation. In endeavouring to tear the mind from a subject to which it has long and closely attached itself, we are almost sure to occasion an irreparable laceration of its structure."

In the third Essay, "On the Fear of Death," the reader will meet with much excellent reasoning, to dispel apprehensions which are, in themselves, more tormenting than the object of dread. The Author deprecates all tendency to encourage despondency; and among the rest, he shows the fatal effects of predictions of death.

"In dangerous maladies, the person in whom there is the least fear of dying, has, other circumstances being the same, the fairest chance to survive. Men, in critical situations, are apt to be overwhelmed by their terrors; they are drowned by their too eager struggles to emerge; they would keep afloat, if they remained quiescent."

The effects of PRIDE on the mind in producing mental derangement, constitute the subject of the fourth Essay; in which we were much pleased with this judicious discrimination in the mode of treating different persons.

"The humbly nervous ought to be treated with the most encouraging respect, and with the most courtier-like attention. We should endeavour, by expressions of an extraordinary regard for them, to supply the want of satisfaction which they are apt to feel with themselves. On the other hand, a haughty imbecility ought to be met by a management that is calculated to depress the patient in his own eyes, and to sober a spirit that may have been intoxicated by draughts of a servile or treacherous adulation."

The next Essay is on "Remorse," which, beyond doubt, is one of the most dreadful of all diseases when it has become fixed in the mind, and the most difficult to cure. But it should be considered, and the author has prudently laid great stress upon the fact, that remorse is not always occasioned by actual misconduct. It is in truth, perhaps, no less frequently the suffering of a tender and upright mind, than of a guilty conscience. Of this we have in addition to some well known cases, the following; which came under the observation of the author himself.

"It is not very long since I had a professional opportunity of knowing something of the morbid history of a man, who had succeeded to a peerage, and an immense estate, by the death of an elder brother, with whom he had not been upon good terms for some years previous to that event. The unfortunate heir to the title and domains so severely reproached himself for that suspension of fraternal amity, with regard to which he was altogether innocent, that he sunk into a profound melancholy, from which I have reason to believe nothing has hitherto been able to rouse him.

"I knew another person, who, although his life had been signalized by the most active and successful exertions in behalf of his fellow creatures, was affected with a despondency, the burden of which was, that he had been all along a useless member of society, and that the talents which had been given him had produced nothing in his hands. Under the influence of this imagination, he expressed a kind of horror as well as shame, at the prospect of giving up a stewardship, the duties of which he had, as he thought, so unfaithfully discharged."

"Not many months ago, I had an opportunity of knowing an instance of the melancholy effect of remorse, where the feeling, although not altogether without foundation, was unduly aggravated by an accidental association of occurrences.

"A young lady was one morning requested by her mother to stay at home; notwithstanding which, she was tempted to go out. Upon her return to her domestic roof, she found that the parent, whom she had so recently disobliged, had expired in her absence. The awful spectacle of her mother's corpse, connected with the filial disobedience which had almost immediately preceded, shook her reason from its seat, and she has ever since continued in a state of mental derangement."

It is well observed, at the commencement of the Sixth Essay, that "an hypochondriac should be a hermit in abstinence, but not in solitude." This subject of seclusion from the pleasures of society is ably treated, and the danger of retirement to those who have been accustomed to business is clearly shewn and supported by proofs. Yet a due caution is introduced with regard to the choice of society; for, as it is properly observed,

"We are not perhaps sufficiently aware that nervous complaints are, through the medium of sympathy, scarcely less infectious than febrile diseases. Amongst many other instances illustrative of this opinion, I particularly recollect the case of an amiable young woman, who, although she had been before remarkable for the uniform cheerfulness and gaiety of her temper, became decidedly, and often deplorably dejected, in consequence of having for a length of time, been domesticated with an elderly friend who was of a desponding and melancholy cast. The contiguous atmosphere of an hypochondriacal, like that of a typhous patient, may, in a certain sense, be said to be impregnated with contagion"

The Seventh Essay is "On excessive Study or Application of the Mind," which, though short, contains some excellent advice to literary gluttons. This Essay is followed by another equally brief, "On Vicissitude as a Cause and Characteristic Symptom of Intellectual Malady." Of the effect of transitions upon the mind, the following instance is given.

"I recollect the case of an unfortunate young man, who became a victim to the disastrous issue of a variety of mercantile adventures. The same blow which deranged his affairs, produced a disorder of his reason. His finances and his facu ties fell together. The phantoms of imagination indeed survived, and seemed to hover over the ashes of his understanding. The demon of speculation, which had before misled his mind, now possessed it entirely. His projecting spirit, which was always more than moderately intrepid, took, in the maniacal exaltation of his fancy, a still bolder and sublimer flight. Some of his schemes reminded me of another madman that I had heard of, who planned, after draining the Mediterranean, to plant it with apple trees, and establish a cyder manufactory on the coast."

The ninth Essay is "On the Want of Sleep," as symptomatic and a cause of mental derangement; in which the author recommends the cold or the warm bath where dietetic opiates have failed.

The next Essay on "Intemperance," contains many valuable cautions with respect to the use and abuse of stimuli; well adapted to make a deep and salutary impression upon the minds of readers in general; but the following is not less deserving the attention of the Faculty.

"Inebriety is not properly confined to the use of fermented liquors. The tipplers of laudanum are sots, although of another sort. There is something peculiarly plausible and seducing in this mode of fascinating the sensations. Opium does not in general, as wine is apt to do, raise a tumult of the feelings, or involve the intellect in clouds; but acts more like oil poured upon a tumultuous sea, which tends to allay the agitation of the billows, and induces an agreeable stillness and tranquillity. Instead of lowering man to a level with the beasts, it often invests him, for a time, with the consciousness and at least fancied attributes of a superior being; but he is soon stripped of his shadowy and evanescent prerogative, and is made to suffer all the horrors and humiliation of a fallen angel. The confessions of many a miserable hypochondriac, who has been in the habit of having recourse to opium for relief, justify this representation from the charge of caricature. Grievous as is the depression which takes place, as the second effect of fermented liquors, that which succeeds to the excitement produced by laudanum, is still more intolerable. It is of course a task less difficult to refrain from the former than the latter, when the latter has been for many years regularly applied to for temporary comfort or support, in a desertion or prostration of the spirits. The late Dr. Heberden was of opinion, that it is more easy to relinquish opium than wine, and therefore, in cases which may seem to require either the one or the other, he recommends the former in preference to the latter. My own comparatively contracted experience would incline me, in the same circumstances, to give different advice.

"I have known only one case, in which an inveterate opium-taker has had resolution enough to dispel the charm which had long bound him to its use. This patient was in the custom of employing it in that concentrated form of the drug, which has received the appellation of the black drop. The dreadful sensations which he experienced for a considerable period, after having refrained from his wonted cordial, he was unable to express, any more than the gratitude which he felt towards his physician, for having strenuously and repeatedly, and at length successfully, urged him to an abstinence from so delusive and bewitching a poison. When opium is employed as a remedy in cases of merely physical disease, it may not be liable to the same objection; although, even in that class of maladies, it ought to be in general reserved for occasions of urgency or peril. When used for a length of time without any considerable intervals, its bad effects upon the constitution will be found to accumulate, whilst its alleviating influence over troublesome and painful symptoms, becomes almost every day less observable."

From the danger of Intemperance, the author proceeds very appropriately to consider the injury occasioned by "the Excess of Abstinence"; for, as he observes, "we may be intemperately abstemious, as well as intemperately luxurious and indulgent. That degree of privation which is unnatural or unreasonable, proves no less destructive than superfluous and superabundant gratification."

This Essay is followed by one on "Morbid Affections of the Organs of Sense"; in which considerable attention is paid to the organ of vision; illustrated by some remarkable cases in the author's own practice. One of these we shall extract.

"During my attendance upon the Finsbury Dispensary, a remarkable instance of dimness of sight occurred, that had for some time previously been gradually approaching towards blindness, which, indeed, had actually taken place in one of the eyes. The patient first perceived the dimness the day after she had been frightened by witnessing a violent paroxysm of epilepsy, with which her husband had been attacked the preceding night. Since that time she had herself become, although not in the least so before, extremely liable to fits, and was apt to fall down insensible upon occasions of the slightest degree of agitation or surprise. Her dimness of sight seemed to consist, not in an injured state of the eye, but in a debility of the nervous system in general, that *appeared* more particularly in that delicate and exquisitely irritable part of it which is destined for the purposes of vision. The capacity of seeing with the eye that was not altogether blind, was intermittent, 'going and coming,' to use her own comparison, 'like the sun when a cloud passes over it.' The patient had likewise been subject to a deafness, that might be traced to the same circumstance as gave rise to her ophthalmic malady. Both symptoms had, in all probability, a common origin in nervous weakness or derangement.

In the Thirteenth Essay, the opinion, or rather vulgar error, is successfully combatted, "that madness, in any of its modifications, arises for the most part from an excess of intellectual vigour."

That "Physical malady may be the occasion of mental disorder," is proved in the next Essay by facts and arguments, while at the same time, the author very properly guards against any leaning towards the doctrine of materialism, which that position might be supposed to favour. We quote from this Essay, with great pleasure, the following reflections on the duty of moderation in scientific pursuits.

"Speculations with regard to the nature of the vital or intelligent principle in man, are involved in so much obscurity, as to allow greater scope for the display of a fertile imagination, than for the sober exercise of the reasoning faculty. The clouds in which this subject is enveloped, the rays of genius may illuminate, but cannot disperse. The unwarrantable boldness and decision with which many are apt to speak upon a question, which, from an incurable deficiency of data, admits of no satisfactory conclusion, argues a more than ordinary imbecility, rather than any superiority of understanding. Genuine intrepidity of every species, is naturally allied to modesty. There is a chaste and sober scepticism. When we profess that there is no moral evidence so immaculately clear, as to preclude all obscuration of doubt, we acknowledge merely the present imperfection and immaturity of our nature. A peremptory positiveness of opinion, as well as a rashness of action, is natural to the ardour and inexperience of youth; but diffidence gradually grows upon declining life. Unlimited dogmatism, in almost every case, affords suspicion of very limited information. In the degree in which our actual knowledge advances, we increase likewise our acquaintance with its comparative deficiency. As the circle of intellectual light expands, it widens proportionably the circumference of apparent darkness.

The Fifteenth Essay contains some good remarks on the polluted atmosphere of the metropolis, from which the author slides gradually into a brief notice of the very old, but not therefore true opinion, "that the gloomy month of November is peculiarly disposing to melancholy, and the favourite season of suicide." This proverbial reproach upon our climate, at that part of the year, is thus dismissed.

"The dark hues of the mind are not in general reflected from the sky; and the preternaturally exalted excitement of mania, soars in general above atmospheric influence. There are cases, indeed, in which the diseased apprehensions of an hypochondriac are relieved or aggravated by the changes of the weather; where, when the sun shines, even his mind seems to be irradiated by its influence, and scarcely a cloud can obscure the face of nature, without at the same time casting a melancholy shade over his speculations."

Having thus far analysed the contents of this excellent work, we are compelled to lay it down for the present, with an intention of considering the remaining Essays in our next Number.

---

